# Historical droughts recorded in extended *Juniperus procera* ring-width chronologies from the Ethiopian Highlands

**DOI:** 10.1007/s00484-020-01863-7

**Published:** 2020-02-01

**Authors:** E. Gebrehiwot Gebregeorgis, I. Robertson, M. Koprowski, L. P. Zhou, P. Gao, A. P. Williams, Z. Eshetu, T. H. G. Wils

**Affiliations:** 1 Department of Ecology and Biogeography, Faculty of Biology and Environment Protection, Nicolaus Copernicus University, Lwowska 1, 87-100 Torun, Poland; 2 Department of Plant Biology and Biodiversity Management, Addis Ababa University, P.O.Box 3434, Addis Ababa, Ethiopia; 3 Department of Geography, College of Science, Swansea University, Singleton Campus, Swansea SA2 8PP, UK; 4 Department of Geography, Peking University, Beijing, 100871 China; 5 Lamont-Doherty Earth Observatory, Columbia University, Palisades, NY USA; 6 Department of Earth Science, College of Life Science, Climate Science Center, Addis Ababa University, Addis Ababa, Ethiopia; 7 Department of Geography, School of Teacher Training for Secondary Education, Fontys University of Applied Sciences, Tilburg, The Netherlands

**Keywords:** Annual tree-rings, Tropical dendrochronology, Radiocarbon dating, Pointer years, The Blue Nile River basin

## Abstract

In the Horn of Africa, little is known about temporal changes in hydroclimate owing to the influence of multiple weather systems, the complex terrain, and the sparse instrumental records. Absolutely dated tree-ring records offer the potential to extend our understanding of climate into the pre-instrumental era, but tree-ring studies in this region, and indeed all of tropical Africa, have been rare largely due to lack of an annual climate cycle that reliably produces annual tree-rings. In this study, 40 cores were obtained from 31 *Juniperus procera* trees growing in the grounds of Ethiopian Orthodox Tewahedo churches in the Gonder region of Ethiopia. The samples were cross-dated using a re-iterative process involving identifying anatomical features from high-resolution images. The tentative ring-width chronologies were revised after the determination of bomb-peak accelerator mass spectrometry radiocarbon dates. Individual series were significantly correlated to the respective master chronologies (r > 0.55; *P* < 0.05), and expressed population signal values ranged from 0.55 to 0.92. Historical drought years were successfully traced in the chronologies by pointer year analysis. This study confirms that *Juniperus procera* growing in areas of unimodal precipitation exhibits annual tree-rings and offers the potential as an indirect measure of past climate.

**Electronic supplementary material:**

The online version of this article (10.1007/s00484-020-01863-7) contains supplementary material, which is available to authorized users.

## Introduction

Ethiopia and its neighboring countries in the Horn of Africa are highly dependent on the rainfall-fed agricultural economy and have suffered from repeated droughts in recent decades (Makombe et al. 2007). The climate of Ethiopia is largely controlled by the seasonal migration of the Intertropical Convergence Zone (ITCZ) and associated weather systems with precipitation originating from both the Indian and Atlantic Oceans. In the boreal summer when the ITCZ moves northwards, most of Ethiopia receives precipitation during the main monsoon-type rainy season from approximately June to September (*Kiremt*). These Kiremt rains support 85–95% of crop growth in Ethiopia, beginning in the southeast and migrating to the northernmost part of the country by mid-July before gradually returning south (Degefu 1988). Some parts of northern and central Ethiopia also experience a less intense secondary rainy season during the spring from approximately February to May (*Belg*). Southern regions of Ethiopia usually experience two distinct rainy seasons as the ITCZ migrates southwards while the east of the country receives very little precipitation at all (Berhanu et al. 2014; Fazzini et al. 2015; Lamb et al. 2018). The determination of Ethiopian hydroclimate on sub-regional scales is hindered by the complex terrain and the sparse network of meteorological stations (Tierney et al. 2013; Nicholson 2014; Nash et al. 2016). Many of the extreme drought events recorded in Ethiopian history have been attributed to the absence of *Belg* and *Kiremt* rains.

As the instrumental climate data do not exceed 70 years and existing global climate models have little skill at recreating seasonal rainfall variations in eastern Africa (Funk et al. 2014; Mwangi et al. 2014), there is need for an indirect measure of past climates to improve our knowledge of the complex climate in this region. Although the hydroclimate of the region has been investigated extensively (Williams and Funk 2011; Williams et al. 2012; Tierney et al. 2013; Lamb et al. 2018), tree-ring records are exceptionally important to consider as potential proxies for inter-annual hydroclimate variability in these region (Fritts 1976; Schongart et al. 2006; Woodborne et al. 2016). The procedure has been widely adopted in temperate regions where trees usually form clearly visible annual tree-rings, a characteristic that is not easy to find in tropics (Worbes et al. 2003; Speer 2010; Pallardy 2013). Tropical dendrochronology is not necessarily a new field since it has been investigated for over a century (Worbes 2002). However, many scientists believed that it would not work in the tropics due to lack of an explicit annual growth cycle with one growing season and one dormant season (Tomlinson and Longman 1981; Détienne 1989; Jacoby 1989). Today, there is a much greater acceptance of tropical dendrochronology, especially in sub-Sahara Africa after the publication of several successful scientific studies (Schweingruber 1992; Wils et al. 2010, 2011a, b; Eshete and Stahl 1998; Worbes 2002; Gebrekirstos et al. 2009; Battipaglia et al. 2015; Gebregeorgis et al. 2018).

The genus *Juniperus* has been used to successfully reconstruct climate over the arid and semiarid zones of the Mediterranean (Touchan et al. 2005, 2007), as well as semi-arid climatic zones of High-mountain Asia (Bräuning 2001), and semiarid, temperate areas of North America (Derose et al. 2016). In this study, we have targeted *Juniperus procera* (Mill.), which has more recently been used to successfully reconstruct climate and Nile river flow in the Horn of Africa (Wils et al. 2010; Mokria et al. 2017, 2018). Cross-dating trees from tropical regions is already a challenge, and is confounded in *J. procera* by the fact that its rings might be confused with density fluctuation or pith flecks that are induced by indistinct seasonality of the climate (Schweingruber et al. 1990) and the fact that it is an endangered species that has limitation of sampling (Farjon 2013). Our study area is located in the Blue Nile River basin, the area known to have been affected by recurrent drought (Keller 1992) but with a low spatial and temporal coverage of climate records (Gasse 2000). The aim of this study is to develop tree-ring width chronologies with a higher dating accuracy than in the existing *J. procera* tree-ring chronologies from the Blue Nile River basin using a combination of more accurate dating methods by increasing the number of samples used for radiocarbon dating and by running pointer year analysis. The resulting ring-width chronologies will lay the foundation for further dendrochronological studies to be conducted in the region.

## Materials and methods

### Description of the study area

**Fig. 1:**
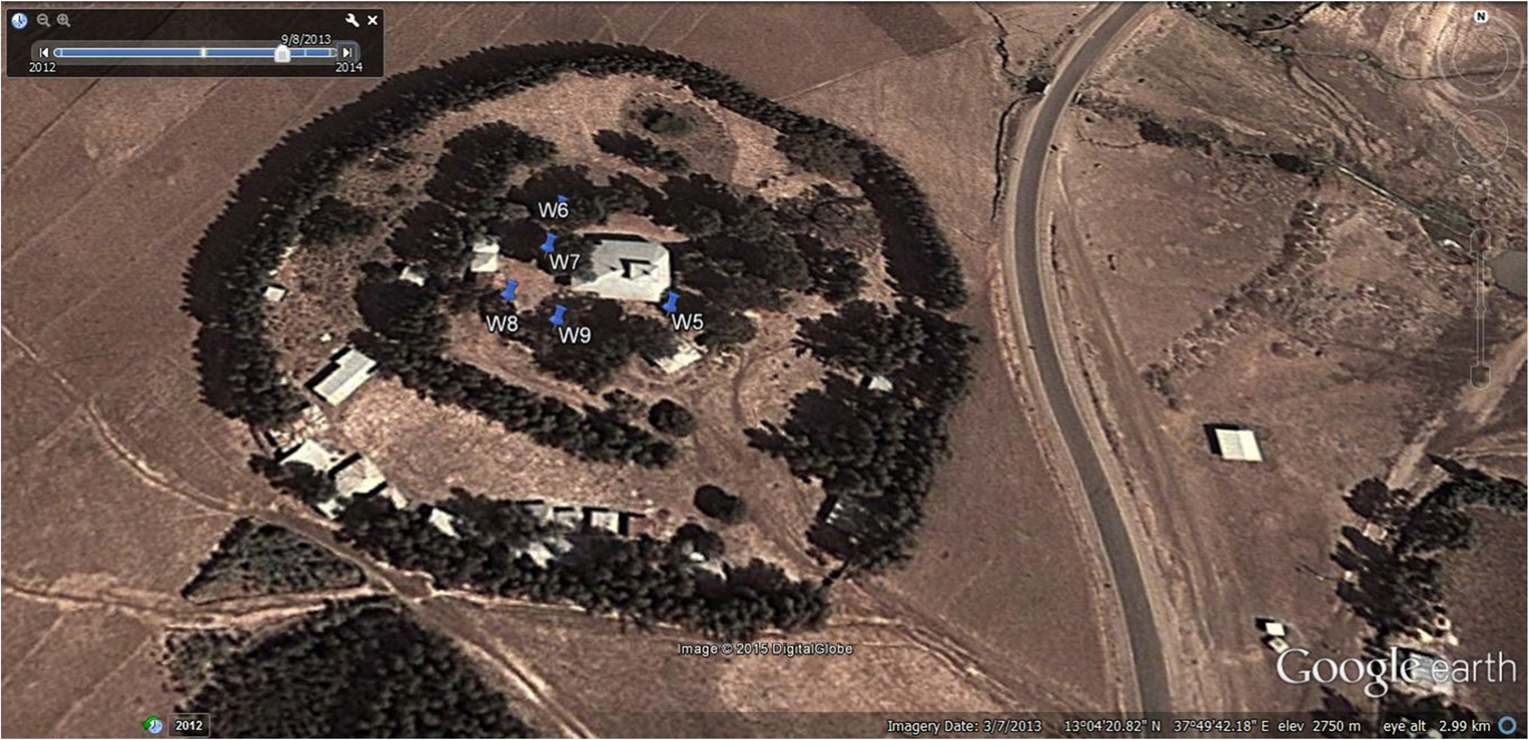
Weken Weybila Maryam church—a representative image of most church grounds of Ethiopian Orthodox Tewahedo churches which preserve trees even if the whole surrounding area becomes deforested. Google Earth (2013)

**Fig. 2:**
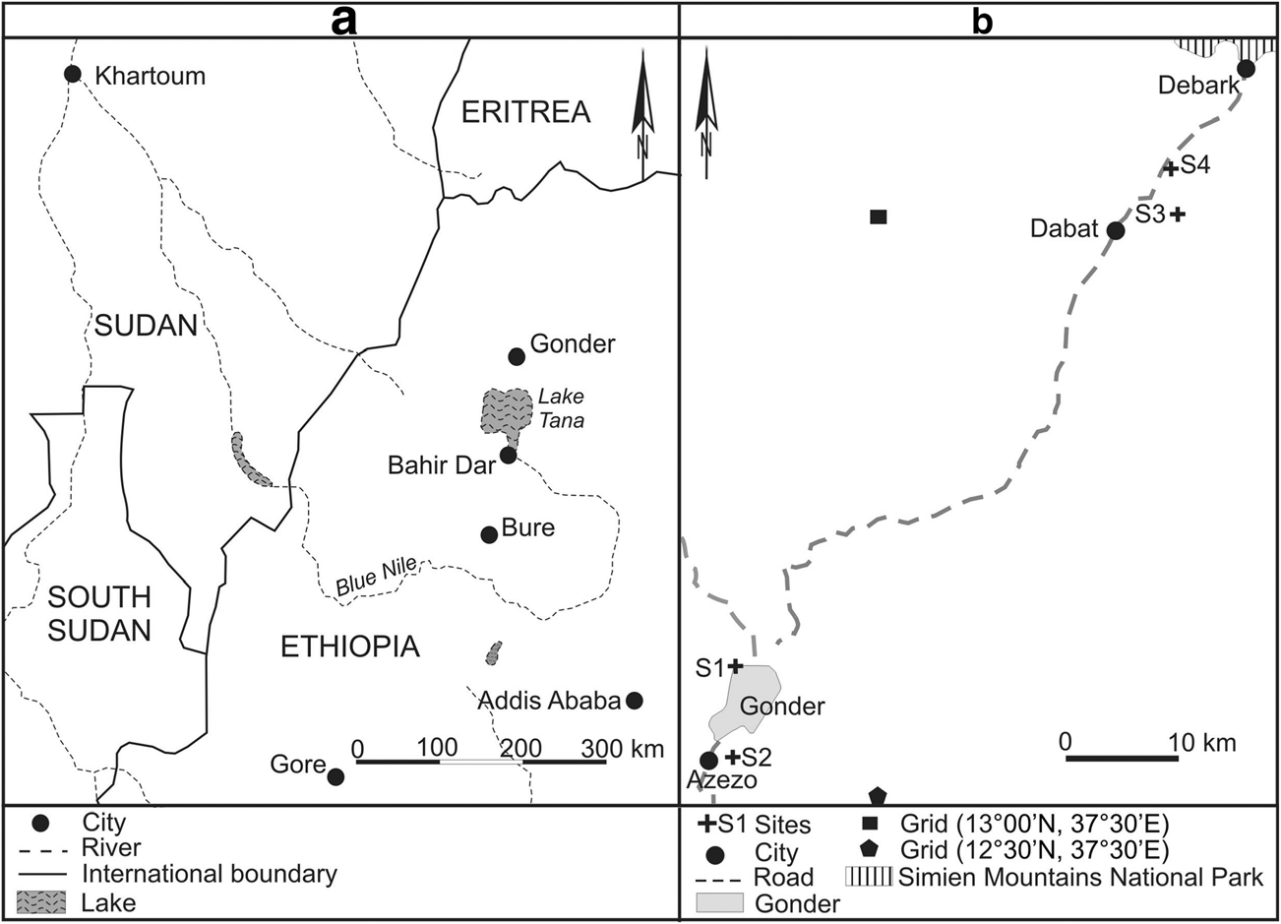
Map of the area of Gonder administrative zone relative to river Blue Nile and neighboring countries (**a**) and the location of the Ethiopian Orthodox Tewahedo church grounds from where the *Juniperus procera* core samples were taken (**b**), i.e., S1 = Gonder Qusquam church, S2 = Rise Adbarat Azezo Teklehaimanot church, S3 = Dabat Dequa Kidane-Mihret church, and S4 = Weken Weybila Maryam church

The core samples were collected from the enclosed compounds of four ancient Ethiopian Orthodox Tewahedo churches (Fig. 1) located in northern Gonder administrative zone of Amhara region of Ethiopia (Fig. 2). Two of the churches, Qusquam (Kuskuam) and Rise Adbarat Azezo Teklehaimanot, are located in Gonder, while the remaining two, Dabat Dequa Kidane-Mihret and Weken Weybila Maryam, are located in the towns of Dabat and Weken, located about 73 and 80 km, respectively, to the north of Gonder. These two towns are classified as the most drought-prone and food-insecure areas of Ethiopia even though they are in the Semien Mountain chain. In the compounds of the churches, trees are usually preserved for centuries, unless they are needed to construct or maintain structures (Alemayehu 2007; Mosissa and Abraha 2018). Church grounds therefore function similarly to arboreta (Fig. 1), preserving old and native trees such as *Juniperus procera.*

### Sampling sites: church grounds

#### Gonder Qusquam church

Located in Gonder city (12°37′20.32″N; 37°26′48.32″E) at an elevation of 2254 m a.s.l. It has a flat terrain with a vegetation comprising freestanding and high-pruned trees, mostly *Juniperus procera*, *Olea europea*, and sparse grasses. The compound is mainly used for spiritual gatherings during mass services and tourism and the back yards for cattle grazing. It is one of the ancient churches in the area believed to be built in the early 1740s.

#### Rise Adbarat Azezo Teklehaimanot church

Located 7.2 km from Gonder and 1 km from Gonder airport (12°32′26.65″N; 37°26′04.73″E) at an elevation of 2750 m a.s.l. It has a flat terrain with poor vegetation cover comprising of few *J. procera* and *O. europaea* trees. The church is surrounded by substantial smallholder farms and sparsely populated settlement. The farming is rain-fed cultivation of crops and vegetables.

#### Dabat Dequa Kidane-Mihret church

Located in a rural area at 80 km distance from Gonder (13°01′42.34″N; 37°50′54.51″E) on a flat terrain at an elevation of 2664 m a.s.l. It has sparsely populated trees of *J. procera* within its compound. It is surrounded by substantial smallholder farmlands where there is a rain-fed cultivation of crops and vegetables.

#### Weken Weybila Maryam church

Located in a rural area, 80 km from Gonder and 7 km southwest of Debark (13°04′20.82″N; 37°49′42.18″E) at an elevation of 2750 m.a.s.l. in the Semien Mountains. The terrain is almost flat and vegetation consists of freestanding *J.* trees with their lower branches cut-off to allow movement of people and sparse grasses. The compound is used for spiritual gatherings and cattle grazing. The church is located in the vicinity of the Semien Mountains National Park (Fig. 2b). The park is home to many endemic plant and animal species and has the highest mountain of Ethiopia, Mount Ras Dashen.

### Climate

**Fig. 3.**
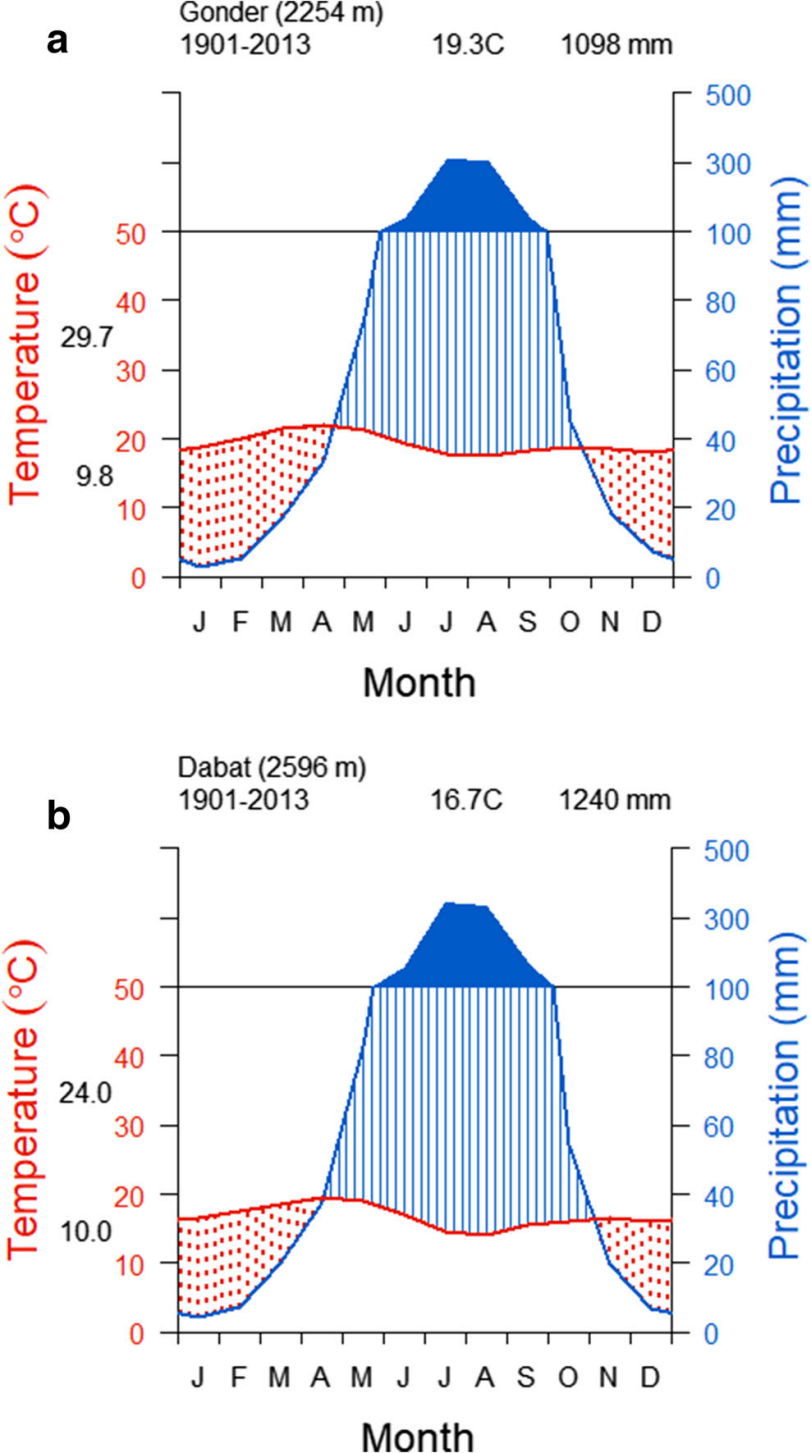
Gridded climate diagrams for the area of Gonder city (**a**), i.e., grid cell 1 (pentagon), and Dabat (**b**), i.e., grid cell 2 (rectangle), over the period 1901-–2013. Each tick mark along the abscissa indicates a month where J represents January, etc. The left ordinate illustrates temperature (°C) with mean daily minimum and maximum temperatures represented by black numbers. The right ordinate indicates monthly precipitation (mm). The blue line represents the precipitation curve with an annual precipitation in Gonder (**a**) and in Dabat (**b**) at the top of the graph. The red line represents the mean monthly temperature, with the average annual monthly temperature at Gonder (**a**) and at Dabat (**b**) indicated at the top of the graph. The solid blue coloured area indicates a period when the mean monthly precipitation exceeds 100 mm. The area coloured with red dots indicates the months with drought stress. Data from CRU database (https://climatedataguide.ucar.edu/climate-data/cru-ts-gridded-precipitationandother-meteorological-variables-1901 (Harris et al. 2014))

The climate data for the study sites for the period 1901–2013 were obtained from the gridded climate database of Climate Research Unit (CRU) (Harris et al. 2014). The spatial coverage of a single grid cell is 50 km^2^, whereas the distance between Gonder and Dabat is about 70 km. Thus, climate data were downloaded for the respective CRU grid numbers, i.e., for the area around Gonder (206,436) and that of Dabat (205,434) (Fig. 2b). Gonder has a unimodal rainfall distribution pattern with the main rainy season occurring between June and September (*Kiremt* in Amharic) and accounting for 81% (897 mm) of the total annual rainfall (1098 mm). July hosts the highest mean precipitation (330 mm) followed by August (307 mm) (Fig. 3). Occasional rainfall in December may occur due to the northern airflow from the Red Sea (Wils 2009). The amount of rainfall in December increases from Gonder northwards while intra-annual variability decreases. The mean monthly temperature varies from 18.0 °C in August to 22.5 °C in April (Fig. 3a). Dabat has mean monthly temperature varying between 18.5 °C in April and 12.0 °C in August (Fig. 3b). Dabat receives its maximum rainfall of 345 mm in July followed by a rainfall of 336 mm in August with a total mean annual rainfall of 1240 mm.

### Sampling and sample preparation

Thirty-one *J. procera* trees were sampled from the four ancient Ethiopian Orthodox church grounds around Gonder administrative zone. The sample size was limited due to the fact that *J. procera* is one of the endangered tree species in this region (Farjon 2013). Using a 5-mm-diameter Haglöf increment borer, two cores per tree were taken at a height of approximately 1.3 m above ground level. Eighteen trees from Gonder Qusquam church, three trees from Rise Adbarat Azezo Teklehaimanot church, four trees from Dabat Dequa Kidane-Mihret church, and six trees from Weken Weybila Maryam church were sampled*.* The cores were rolled in moisture absorbent papers to prevent decay and placed in rigid plastic conduits to protect them from physical damage. The cores were air dried and sanded with progressively finer grades of abrasive paper (Orvis and Grissino-Mayer 2002).

### Tree-ring boundary detection, cross-dating, and tree-ring width measurement

The main purpose of cross-dating was to find the best match between annual growth patterns to enable the establishment of a site chronology with absolute confidence in the assigned dates (Friis 1992; Wils et al. 2011a). Cross-dating supported by the determination of wood anatomical features (Stokes and Smiley 1968) leads us to focus on pointer year analysis which may be better suited to these challenging samples, where a re-iterative process is required (Wils et al. 2011a).

Each tree-ring was photographed using an opto-digital microscope (Leica M205 C) with camera (Leica DFC 495). For samples with features that were difficult to identify (Fig. 5b), the wood was marked with a scalpel and freehand sketches were made to illustrate the area of concern. This procedure helped with the identification of false and missing rings (de Micco et al. 2016), which in turn strengthened the re-iterative process of cross-dating (Wils et al. 2011a).

**Fig. 4:**
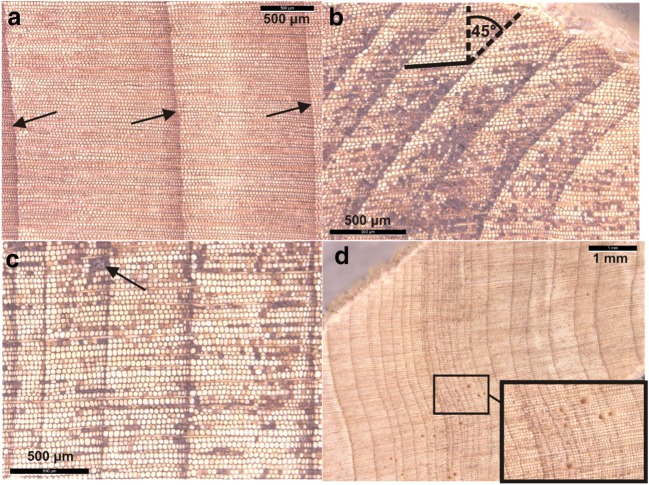
Various types of *Juniperus procera* tree-rings: normal (**a**) arc-shaped (not perpendicular to the rays; **b**), showing dark brownish tracheid lumens (of the heartwood predominantly; **b**, **c**) and micro-rings (**d**)

The sample cores were scanned using (Epson Perfection V700) photo scanner at a resolution of 1200 dpi. The widths of the tree-rings were measured on the scanned images using the program, CooRecorder V7.5 (Larsson 2003a). Measurements were determined parallel to medullary rays. In case of the arc-shaped nature of some of the tree-rings (Fig. 4b), tree-rings were measured at the centers of the arcs they formed (Bryukhanova and Fonti 2013).

The measured tree-ring width series were saved in “.pos” file format for each radius and imported to the cross-dating program, CDENDRO V7.5 (Larsson 2003a). Within tree cross-dating was conducted by visual comparison of growth curves (Pilcher 1990) and statistical measures, i.e., cross-correlation and t-values, where each pair of cores were tested individually. In this process, samples were frequently re-measured, false and double rings were excluded through the process of trial-and-error frequently referring to the pictures and the notes prepared during initial tree-ring identification. After assuring that all the pairs of cores were cross-dated, the mean tree-ring width values were calculated. As up to two cores per tree were collected and then after excluding some of them for their low coherence with the rest of the sample, 40 cores from 31 *J. procera* trees were included in the final site tree-ring chronologies.

Age and physical disturbance related trends were removed from individual ring-width measurements by fitting negative exponential curve (Cook and Kairiukstis 1990). Each detrended series was then standardized using a cubic smoothing spline with a frequency cut-off of 50% and rigidity of two-thirds of the total series curve length within the Dendrochronology Program Library in R (dplR) (Bunn 2008) (Cook and Peters 1981). The adequacy of sample size was checked by computing expressed population signal (EPS) (Wigley et al. 1984) in R (dplR) (Bunn 2008). Other summary statistics which evaluate the validity of the tree-ring chronologies such as mean sensitivity (MS) (Douglass 1920) and mean inter-series correlation (Cook and Kairiukstis 1990) were calculated. Similarity of site tree ring chronologies was tested by running a correlation calculus based on P2YrsL: proportion of last 2 years growth LIMITED in C Dendro 9.3.1 that gives their correlation and *t-*test values (Larsson 2003b).

### Radiocarbon dating

As a part of the re-iterative process of cross-dating (Wils et al. 2010, 2011a), radiocarbon dating was employed to test the developed provisional ring-width chronologies. Seven samples from cores with the highest inter and intra tree correlations, i.e., samples 14B and 17A from Gonder Qusquam church and the core sample 8A, from Weken Weybila Maryam church, were selected for accelerator mass spectrometer (AMS) radiocarbon dating conducted at Peking University, China. Bomb-peak radiocarbon dating may be used to determine elevated ^14^C values originally derived from atmospheric carbon dioxide to date samples to the nearest year from approximately AD 1955 to present (Vogel et al. 1989; Campana and Jones 1998; Kaplan 2003; Reimer et al. 2004; Robertson et al. 2004; Andreu-Hayles et al. 2015). α-Cellulose was isolated from annual late-wood slivers using standard techniques (Loader et al. 1997; Rinne et al. 2005) and homogenized using a Hielscher ultrasonic probe (Laumer et al. 2009) to yield a homogenous sample. The sample was combusted to carbon dioxide and reduced to graphite on an iron catalyst using the zinc reduction method (Xu et al. 2007). ^14^C/^12^C and ^13^C/^12^C ratios were determined using the compact ^14^C AMS system developed by the National Electrostatics Corporation based upon the Model 1.55SDH-1 Pelletron accelerator with a terminal voltage of 0.6MV (Liu et al. 2007). Values were corrected for isotopic fractionation using the AMS-derived ^13^C/^12^C ratio and converted to fraction modern ^14^C (F^14^C) values (Stuiver and Polach 1977; Reimer et al. 2004). Calibration of dates was achieved using the Calibomb function in Calib 7.1 (Reimer et al. 2004; Stuiver et al. 2017) using atmospheric radiocarbon measurements from the Northern Hemisphere Zone 3 (Hua et al. 2013) supported by the limited atmospheric radiocarbon measurements from Debre Zeit in Ethiopia (Nydal and Lövseth 1996). A smoothing function of 1 year was selected to minimize inter-annual variability (Stuiver et al. 2017). As part of the re-iterative dating process, the AMS radiocarbon dates were used to correct ring-width dates where there was a deviation from the tentative initial chronology.

### Pointer year analysis

After developing the final site chronologies, pointer year analysis was conducted where abrupt negative or positive changes in tree growth were used to cross check the validity of dating and indicate extreme events recorded in the chronologies (Schweingruber et al. 1990; Lebourgeois et al. 2005; Neuwirth et al. 2007). To identify pointer years, the indexed site tree-ring chronologies were imported in to the program, Weiser (Gonzales 2001), to allow the identification of extreme years. Considering the sensitivity of *J. procera* to climate at the Blue Nile River basin (Wils et al. 2010), the pointer year window width was set to 5 years with the pointer year interval set to 75 years as recommended for such sensitive tree species (Gonzales 2001). The pointer year statistics analysis accounts the variation within a sample of trees. It only reveals signals common to most trees and is less affected by outliers. It is applied on normalized series and it sets negative and positive threshold values of standard deviation for negative and positive pointer years respectively (Meyer 1998–1999). Thus, the negative values of the pointer year statistics are sourced from the negative values of standard deviation.

The pointer year statistic can be calculated based on the following equation:

$$ P{S}_i= mean\left( RW{I}_i\right)\ast \mathit{\log}\left({n}_i\right)/ stdev\left( RW{I}_i\right) $$

where *PS*_*i*_ is an indexed value in a year *i*; *i* is the year of focus; *mean* (*RWI*_*i*_) = arithmetic mean of the year ring width index (RWI) among *n*_*i*_ samples in the year *i*; log (*ni*) is a common (base ten) logarithm of the number of tree-ring samples in the year *i* and *stdev* (RWI*i*) is the standard deviation of the tree-ring width of *n*_*i*_ samples in the year *i* (Meyer 1998–1999).

In the sub-Saharan climate, the most commonly occurring climate-induced catastrophe is drought. Thus, the analysis focused on the negative pointer years in order to compare them alongside the list of historical drought years (Webb et al. 1991; Comenetz and Caviedes 2002; Viste et al. 2012). The list of historical drought years (Degefu 1988) was crosschecked against the negative pointer years identified within the time period AD 1757–2013. Where the pointer year values fell between − 1 and − 3, the year was classed as likely to be a drought-linked pointer year. The identification of negative pointer years confirmed cross-dating. To avoid circularity, the historical drought data were regarded as independent and not used to correct the ring-width chronologies.

## Results

### Tree-ring identification, properties, and cross-dating

**Fig. 5:**
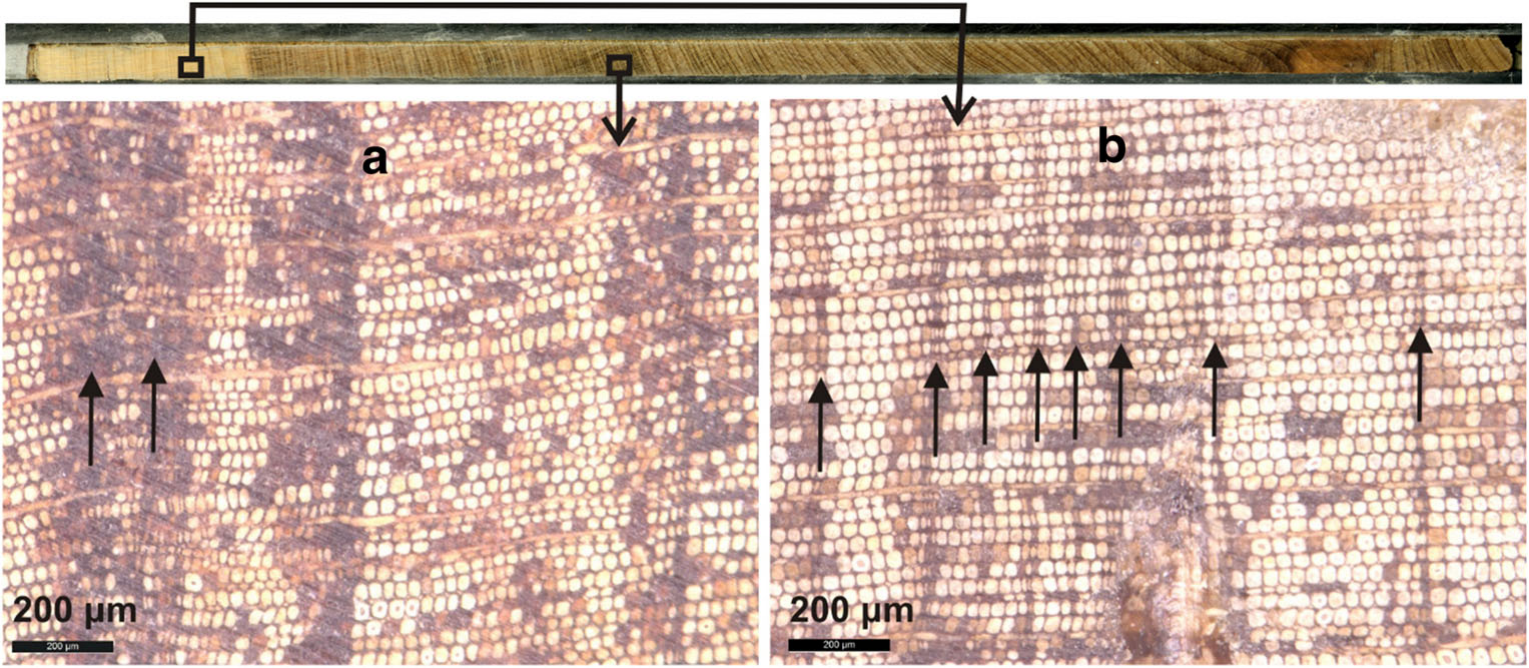
Micro-rings observed during the study (Gonder Qusquam church 17A year’s 1937 to 1943). The two arrows in the picture on the left demonstrate the micro-rings with dark brownish shade, which made distinguishing tree-ring boundaries difficult (**a**). The micro-rings can be confused with false rings (**b**)

Among the frequently observed tree-ring anatomy features of *J. procera* in this study are extremely narrow micro-rings (Figs. 4d and 5a) (Bryukhanova and Fonti 2013); in tree-rings close to the pith where tree-ring boundaries were dominated by dark brown stains, which made the entire tree-ring resemble late-wood (Fig. 5a); and the presence of many dark dots and coloured cells tangentially distributed throughout the tree-rings (Figs. 4b and 5a).

Moreover, during the study micro-rings with damaged or dark brownish look of tracheids, mostly on the tree-ring boundaries were observed as demonstrated in Figs. 4d and 5b).

### Radiocarbon dating

For sample 14B from the Gonder Qusquam church, three samples were selected for radiocarbon dating (Table 1). The date of the inner sample initially allocated to the year AD 1965 (radiocarbon sample QAS-3619) was confirmed. Calibration of the “middle” sample initially allocated to AD 1976 (radiocarbon sample QAS-3620) gave two possible dates AD 1976–78 and AD 1963, at two sigma errors. Through a process of elimination, the date of AD 1963 can be rejected as this sample cannot be younger than QAS-3619. Similarly, calibration of the outer sample initially allocated to AD 2001 (radiocarbon sample QAS-3622) gave two possible dates AD 2000–2004 and AD 1958 at two sigma errors. As this outer sample cannot be older than QAS-3619 and QAS-3620, the approximate date of AD 2001 was confirmed.

**Table 1:** Calibration of radiocarbon dates using the Calibomb function in Calib 7.1 (Reimer et al. 2004; Stuiver et al. 2017). The dates were normalized to δ^13^C = -25.0‰ and reported with the conventional one sigma (Stuiver and Polach 1977) and two sigma (2σ) errors. Atmospheric radiocarbon measurements from the Northern Hemisphere Zone 3 (Hua et al. 2013) were used. A smoothing function of one-year was selected to minimize inter-annual variability

Core	Laboratory no.	Provisional age (AD)	Most probable age (AD)	F^14^C	1σ (F^14^C)	Calibrated dates (2σ range); probability
8A	QAS-3617	1965	1964–1966	1.673127	0.0064	[cal AD 1963.80: cal AD 1964.79] 0.374
						[cal AD 1965.46: cal AD 1966.95] 0.626
	QAS-3623	2006	2006–2008	1.057803	0.0032	[cal AD 1957.62: cal AD 1957.85] 0.062
						[cal AD 2005.70: cal AD 2008.15] 0.938
14B	QAS-3619	1965	1964–1966	1.692033	0.0049	[cal AD 1964.23: cal AD 1966.43] 1.000
	QAS-3620	1976	1976–1978	1.345576	0.0050	[cal AD 1962.61: cal AD 1962.78] 0.074
						[cal AD 1976.01: cal AD 1978.16] 0.926
	QAS-3622	2001	2000–2004	1.084570	0.0036	[cal AD 1957.89: cal AD 1958.15] 0.055
						[cal AD 2000.46: cal AD 2003.74] 0.945
17A	QAS-3618	1965	1964–1965	1.723082	0.0048	[cal AD 1964.57: cal AD 1965.90] 1.000
	QAS-3621	2001	2002–2006	1.073875	0.0031	[cal AD 1957.78: cal AD 1958.03] 0.048
						[cal AD 2002.35: cal AD 2005.83] 0.952

**Fig. 6:**
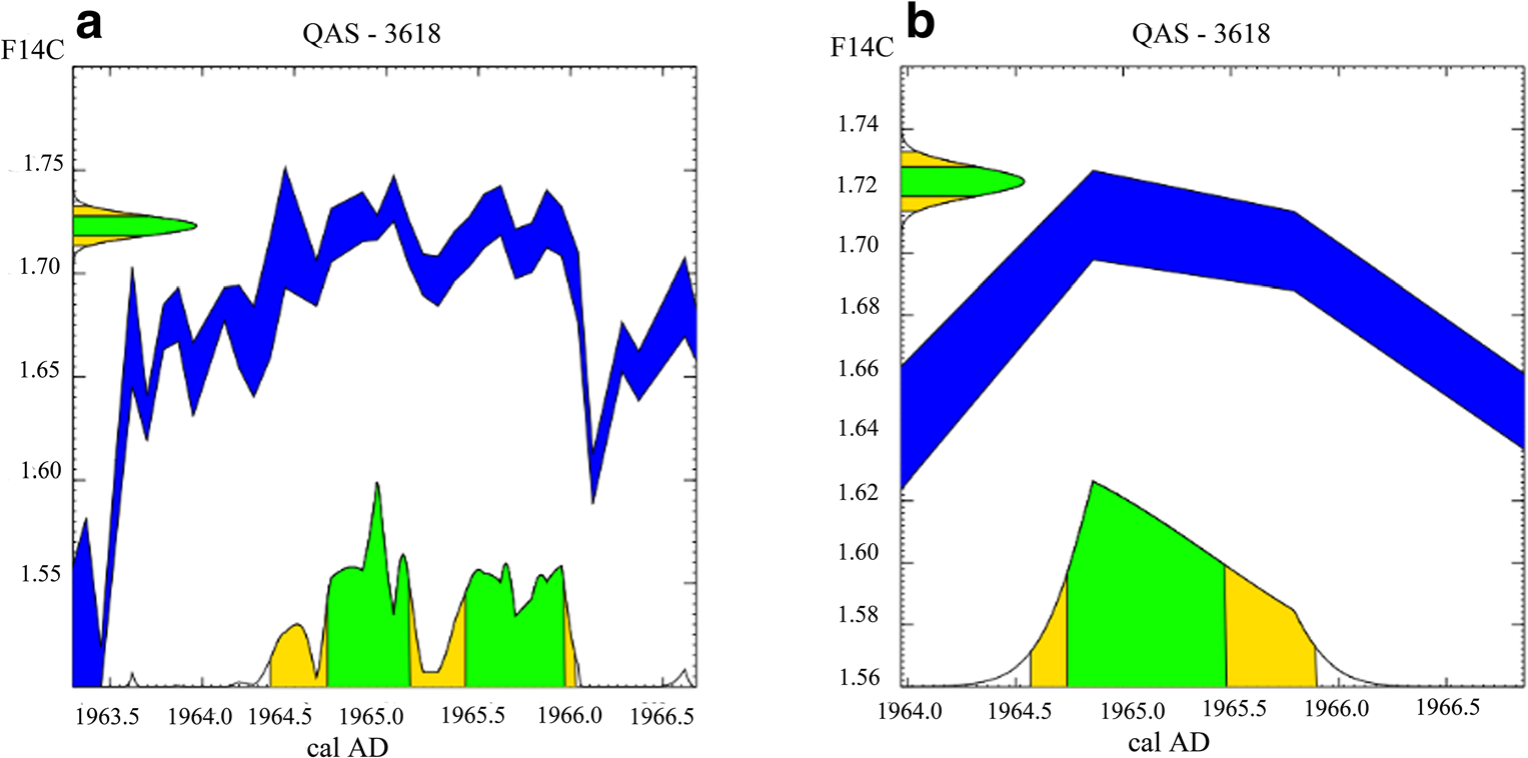
Calibration of radiocarbon dates using the Calibomb function in Calib 7.1 (Reimer et al. 2004; Stuiver et al. 2017) with F14C = 1.7230 ± 0.0048 (*ordinate*) (**a**, **b**). Atmospheric radiocarbon measurements from the Northern Hemisphere Zone 3 (Hua et al. 2013) were used without smoothing (blue line) (**a**) and a smoothing function of oneyear was selected to minimise inter-annual variability (blue line) (**b**). The 2σ calibrated probability ranges (green shading) are shown on the calendar axis (cal AD) (abscissa) (**a**, **b**). The 2σ calibrated probability ranges (green shading) are shown on the calendar axis (cal AD) (abscissa) (**a**, **b**)

For sample 17A, also from the Gonder Qusquam church, two samples were selected for radiocarbon dating (Table 1). The date of the inner sample, initially allocated to the year AD 1965 (radiocarbon sample QAS-3618), was confirmed by calibrating the F^14^C values using Northern Hemisphere zone 3 (NH3) atmospheric radiocarbon measurements with no smoothing (Fig. 6a) and a smoothing filter set to 1 year to minimize inter-annual variability (Fig. 6b; Stuiver et al. 2017). Using a similar approach, the outer sample, initially allocated to the year AD 2001 (radiocarbon sample QAS-3621), gave two possible dates AD 2002–2006 and AD 1957–58 at two sigma errors. As this outer sample cannot be older than QAS-3618, the date of AD 1957–58 can be rejected. However, the AMS radiocarbon date for the outer sample appeared to be offset by 1 year. Re-inspection of the samples and wood anatomical features revealed a false ring that was removed from the ring count, and the chronologies were re-calculated taking this change into account.

Two samples were selected for radiocarbon dating from core 8A from Weken Weybila Maryam church (Table 1). Adopting the same procedure, the inner sample initially allocated to the year AD 1965 was offset by 1 year. Re-inspection of the cores and images revealed that the presence of a false ring that was either an intra-annual density fluctuation (IADF) or a dating error and the initial tentative chronology were subsequently revised.

### Tree-ring width measurement statistics

**Fig. 7:**
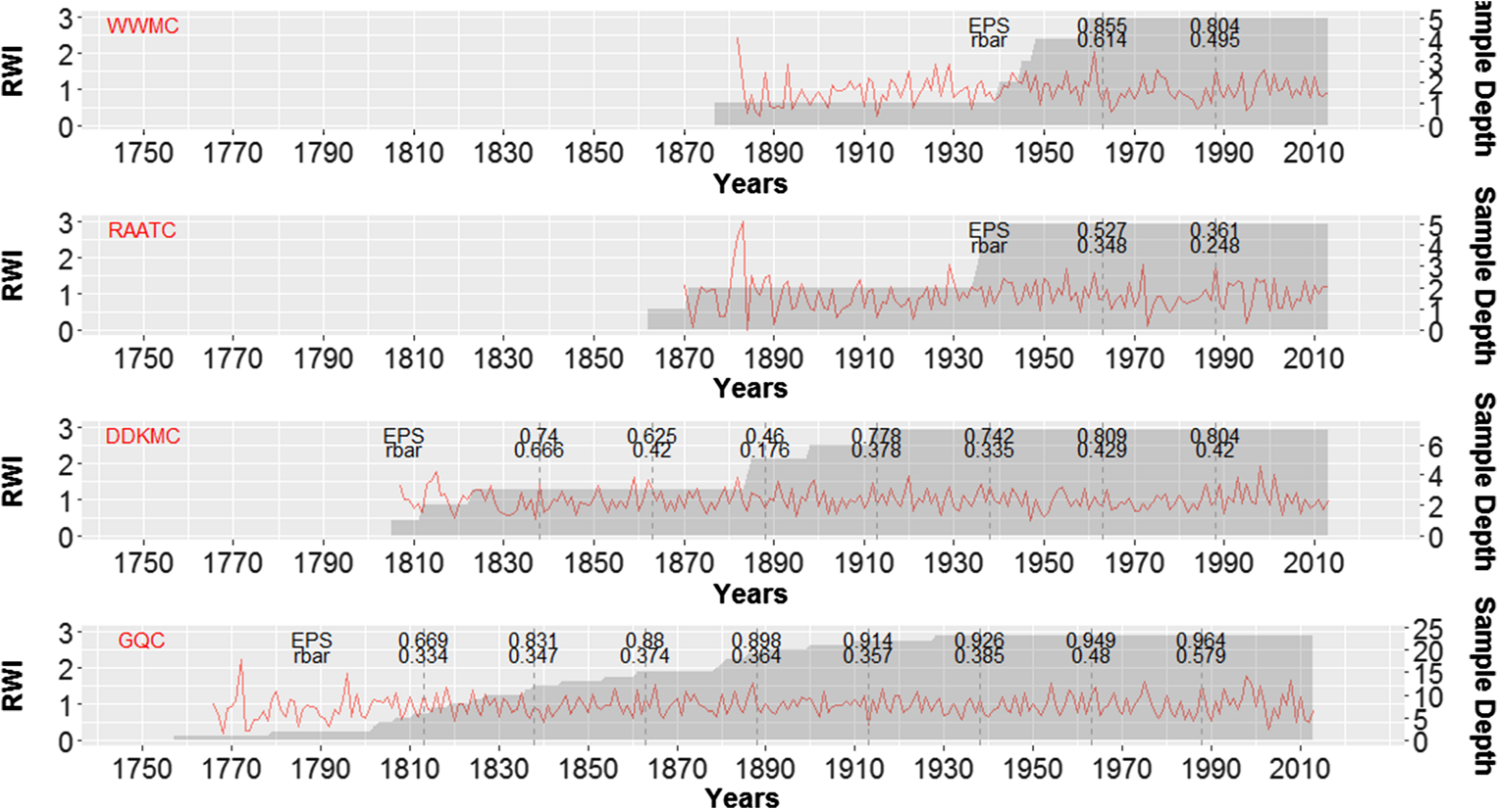
Residual chronology (RWI) of each site, i.e., Gonder Qusquam church (GQC), Rise Adbarat Azezo Teklehaimanot church (RAATC), Dabat Dequa Kidane-Mihret church (DDKMC), and Weken Weybila Maryam church (WWMC) with their sample depths (Shaded area). Their respective EPS and rbar for 50-year window with 25-year lag where dashed line shows the middle of the window and EPS and rbar values represent 50-year window with 25 years before and after dashed line

Tree-ring data of 40 cores from 31 trees were successfully cross-dated; statistically characterized and site chronologies were developed (Table 2; Fig. 7). The oldest samples were from Gonder Qusquam church and the youngest ones were from Weken Weybila Maryam church. The mean sensitivity varied across all the four sampling sites. The lowest value was observed on the samples collected from Dabat Dequa Kidane-Mihret church and the highest ones were from Rise Adbarat Azezo Teklehaimanot church and Weken Weybila Maryam church. The correlation of each tree-ring series with their respective master chronologies per site was strong (r > 0.55; *P* < 0.05) in all the four chronologies. The average first order autocorrelation was high (r > 0.40; *P* < 0.05) (Wigley et al. 1987) in all chronologies (Table 2). The expressed population signal (EPS) is a measure of the degree to which a chronology is representative of a perfect chronology (Cropper 1982; Wigley et al. 1984). In other words, it is a measure of similarity of a chronology and a hypothetical chronology based on all trees in a population (Briffa et al. 1990). In this study, EPS values for the chronologies from Gonder Qusquam church ranged between 0.55 and 0.92 (Briffa and Jones 1990; Wigley et al. 1984).

**Table 2:** Summary statistics for the *Juniperus procera* site ring-width chronologies

Variables (unfiltered)	Gonder Qusquam	Dabat Degua-Kidanemihret	Rise Adbarat Azezo Teklehaimanot	Weken Weybila Maryam
Expressed population signal (EPS)	0.919	0.767	0.550	0.761
Age (time period)	86–257 (1758–2013)	102–209 (1804–2013)	77–152 (1861–2013)	137 (1878–2013)
Mean sensitivity	0.400	0.366	0.454	0.426
Auto correlation	0.481	0.546	0.638	0.466
Correlation with master	0.633	0.562	0.551	0.647
Standard deviation of ring width index	0.695	0.933	1.135	1.008
Number of trees	18	6	3	4
Number of cores	23	7	5	5
Residual ring-width index	0.997 (0.156–2.063)	1.003 (0.496–2.177)	0.980 (0.248–2.849)	0.973 (0.028–3.738)
Standard ring-width index	0.997 (0.110–2.070)	0.994 (0.436–2.360)	0.978 (0.178–3.482)	0.973 (0.028–3.738)

The chronologies from all the four sites showed similar patterns and significantly correlated to each other (*P* < 0.05) (Table 3). The narrowest as well as the widest tree-rings were observed on samples from Weken Weybila Maryam church (Table 2). On the other hand, looking at the general trend, it is noticeable that the oldest samples and of the most uniform growth were observed at Gonder Qusquam church. The second oldest samples were from Rise Adbarat Azezo Teklehaimanot church, and they had the second widest tree-rings on average, following samples from Dabat Dequa Kidane-Mihret church (Fig. 7; Table 2).

**Table 3:** Correlation coefficient and *t*-test values of the four site chronologies, i.e., S1 = Gonder Qusquam church, S2 = Rise Adbarat Azezo Teklehaimanot church, S3 = Dabat Dequa Kidane-Mihret church and S4 = Weken Weybila Maryam church

Dated		2013	2013	2013
Corr TTest	S1	S2	S3	S4
S1		0.35	0.35	0.46
S2	4.6		0.28	0.71
S3	5.4	3.6		0.41
S4	6.0	11.8	5.2	

### Pointer and drought years

**Fig. 8:**
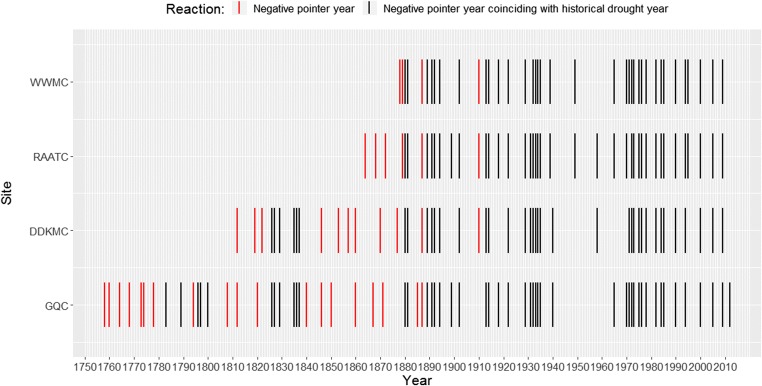
Presence of historical drought years appearing as negative pointer years (black bars) and that of non-drought negative pointer years (red bars) in the four-site chronologies developed in this study. Gonder Qusquam church (GQC), Rise Adbarat Azezo Teklehaimanot church (RAATC), Dabat Dequa Kidane-Mihret church (DDKMC), and Weken Weybila Maryam church (WWMC)

The list of negative and positive pointer years was obtained from the computation by WEISER (Gonzales 2001). The output appears in a tabular form showing the negative and positive pointer years to the left and right of the neutral years (0 values) with their equivalent degrees of intensity ranging from − 3 to 3. The list of historical drought years compared alongside the negative pointer years in each site chronologies is shown (Fig. 8). On average, about 85% of correspondence between the list of negative pointer years and historical drought years was observed over the four site chronologies. This in turn means there are negative pointer years which are not drought years.

In a similar manner to other studies, these trees recorded historical drought years (Verschuren et al. 2000; Nicholson 2001; Legesse et al. 2002; Lamb et al. 2007; Umer et al. 2007). However, it is important to realize that it does not necessarily mean that all the negative pointer years were only recorded during the historical drought years (Neuwirth et al. 2007).

## Discussion

### Tree-ring formation and cross-dating

Tree-ring series of 40 cores from 31 *Juniperus procera* trees from four church grounds at Gonder and Dabat in Ethiopia were successfully cross-dated and confirmed by AMS radiocarbon dating and incorporated into four separate site chronologies (Fig. 7; Table 2). Subsequently, historically known drought years were traced in the chronologies (Fig. 8). Similarly, in our earlier study on different sample trees, cross-dated tree-ring chronologies of *J. procera* at the Blue Nile River basin were also built at Gonder where historical drought years were used as indicator years to assist the re-iterative cross-dating procedure of *J. procera* at Gonder. Similar results have also been found at lake Tana and Gonder areas, i.e., at the Blue Nile River basin by Mokria et al. (2017, 2018), who constructed a multi-century *J. procera* ring-width chronology from living and dead trees to reconstruct precipitation (Mokria et al. 2017) and the flow dynamics of the river Blue Nile during AD 1784–2014 (Mokria et al. 2018).

The sanding method employed contributed towards the successful identification of false rings by enhancing the visibility of individual tracheids and degree of sharpness of the return to earlywood as reported by Vaganov et al. (2009). The re-iterative approach to dating shared many characteristics with skeleton plotting, with ring identification, marking, and the graphical comparison of ring width patterns being used in a similar manner until the time-series attained the minimum acceptable statistical value (Cropper 1979; Swetnam et al. 1985; Cook and Holmes 1986).

Cross-dating the *J. procera* tree-rings at Gonder was a complex and challenging task due to the unusual tree-ring and tracheidal properties, including frequent wedging, missing rings, and false rings (Fig. 5). Similar challenges have been reported by numerous studies conducted in tropical and Mediterranean tree species (Schweingruber et al. 1990; Cherubini et al. 2003; Campelo et al. 2016; Nabais et al. 2014; Kurz-Besson et al. 2016), including *J. procera* (Couralet et al. 2007; Wils et al. 2011a). This challenge is more pronounced for conifers grown in semiarid regions (Schweingruber 1988) such as in the Gonder region (Couralet et al. 2005) (Fig. 3). Further, this region is tropical, and tropical climates promote the formation of non-annual rings, i.e., double and missing rings are common (Tomlinson and Longman 1981; Wils et al. 2011b).

The arc-shaped nature of some tree-rings of *J. procera* has also resulted in non-perpendicular intersection between tree-rings and rays (Fig. 4b), which is uncommon (Speer 2010; Schweingruber et al. 1990). The appearance of curling-up (twisting) stems of *J. procera* trees must have contributed to this observation. It may have also been created by external forces of gravity and wind (Schweingruber et al. 1990).

The highly frequent appearance of micro-rings observed on the *J. procera* samples (Figs. 4d and 5b) can easily be confused with false rings. False ring occurrence is common in many trees, especially conifers (Schweingruber et al. 1990; Campelo et al. 2016). Thus, recent studies have suggested the adoption of independent and reliable dating techniques such as radiocarbon dating to help improve the reliability of tropical tree-ring chronologies (Herrera-Ramirez et al. 2017; Worbes et al. 2017). Considering these challenges, the analysis of disc samples would have been better (Worbes 2002), but *J. procera* is an endangered tree species (Azene 2007) and destructive sampling of trees was not allowed from the grounds of Ethiopian Orthodox Tewahedo churches.

### Radiocarbon dating

Radiocarbon dating was helpful in validating the tentative dates obtained by cross-dating (Table 1), and this technique has been utilized elsewhere (Hua et al. 1999; Fichtler et al. 2004; Biondi et al. 2007). The elimination of the least probable date from the prior and post bomb-peak period (Tans 1981; Vogel et al. 1989; Levin and Kromer 1997) increased the reliability of the overall dating process (Biondi and Fessenden 1999; Andreu-Hayles et al. 2015). Although the calibration of radiocarbon dates uses the closest available regional atmospheric ^14^C data to reduce the influence of external factors such as fossil fuel combustion (Levin and Hesshaimer 2000; Levin et al. 2003), the atmospheric radiocarbon concentrations measured from Debre Zeit in Ethiopia were also utilized to confirm results (Nydal and Lövseth 1966). Most of the radiocarbon dates exactly matched the tentative dendrochronological calendar dates (Table 1). Similar results have been observed for trees growing in tropical environments. *Pinus rigida* from Madidi National Park in Bolivia showed strictly annual tree-rings and their dendrochronological dates exactly matched with the results of the radiocarbon dates (Andreu-Hayles et al. 2015). In the wet tropical forests of Central Africa, it was reported that four out of five tree species tested showed an annual periodicity in their tree-rings (Groenendijk et al. 2014).

On the other hand, differences were observed between radiocarbon dates and tentative ring-width dates on radiocarbon samples QAS3621 and QAS3617 from tree-ring samples, 17A-2001 and 8A-1965 respectively (Table 1). These differences were caused due to missing rings and the presence of false rings. This is also consistent with the results of several tropical radiocarbon studies where differences were observed between radiocarbon dates and tentative dendrochronological dates. For example, the initial *J. procera* chronology from the Blue Nile River basin showed a 1-year difference from bomb radiocarbon dates and the chronology was revised (Wils et al. 2010). In another tropical study, Linares et al. (2017) showed that in some years, the radiocarbon dating revealed 7–12 missing rings between two consecutive dendrochronologically dated tree-rings. False rings can also lead to incorrect dating, as several false rings can occur within 2 years. Worbes et al. (2017) investigated 27 tropical peat swamp forest trees in Kalimantan, Indonesia, and found that some species exhibit multiple tree-rings per year. Similarly, Wils et al. (2009) used bomb-peak radiocarbon dating to show that *J. procera* from Doba forest, northern Ethiopia, had multiple tree-rings per year, and Herrera-Ramirez et al. (2017) found that *Prioria copaifera* from Atrato River, Colombia, formed multiple tree-rings in certain years, which made radiocarbon dating essential for validating the annual periodicity of tree-rings especially in the tropics.

### Tree-ring width analysis

The tree-ring width chronology from Gonder Qusquam church was built from living trees and is one of the longest chronologies from tropical Africa (Mokria et al. 2017, 2018; de Ridder et al. 2014; Schongart et al. 2006; Fichtler et al. 2004; Gebrekirstos et al. 2008). As the tree-rings are annual, their age must be at least equal to the number of their tree-rings and older as several rings are missed when sampling at a height of 1.3 m above the ground (Worbes 1999).

Although the sample size is relatively small, the high degree of temporal autocorrelation among ring widths (Table 2) indicates that the tree-ring width is influenced by the climate of several years (Carrer and Urbinati 2004). Generally, in the tropics, the main growth determinant climatic variable is precipitation (Worbes 2002; Cleaveland et al. 2003; Brienen and Zuidema 2005). The autocorrelation in the tree-ring width chronology from Rise Adbarat Azezo Teklehaimanot and Dabat Dequa Kidane-Mihret (Table 2) church exceeded the acceptable thresholds (Cook 1985). Additionally, evergreen needles involving in photosynthesis for multiple years might also have contributed for the higher autocorrelation (Fan et al. 2009). At these sites, the trees grew in church grounds where there was considerable spacing between trees and higher ground disturbance by cattle grazing that could have increased autocorrelation (Holmes 1983). At Gonder Qusquam and Weken Weybila Maryam, the autocorrelation was lower reflecting lower ground disturbance as the churches have wider compounds even though they are located closer to city and densely populated areas. A similar result was observed on the drought-sensitive tree-ring chronologies from Kyrgyzstan and China indicating low effect of previous years’ climate on the current year’s growth (Wang et al. 2017).

The EPS values of the four tree-ring width chronologies of *J. procera* varied among sites, and it showed direct proportionality to sample depth and inverse proportionality to elevation of sampling sites (Table 2). A similar result was found by Mérian et al. (2013). The highest value of EPS was obtained for the Gonder Qusquam church chronology, which was partly related to the higher sample depth. Many studies have misinterpreted the concept and purpose of EPS and even set a minimum requirement of 0.85 (Buras 2017). But, there is no minimum or optimum value to be stated as a statistical requirement of a chronology to be accepted or to do not (Wigley et al. 1984; Buras 2017). Thus, an EPS value of below 0.85 cannot necessarily be the only reason to exclude site chronologies from further dendroclimatic analysis. The chronology at each site significantly correlated with the master chronology which is composed of all trees per site (Table 2) indicating a high degree of common forcing. Moreover, these chronologies were developed using expensive and labor-intensive methodologies with an intension of compromising the lower sample depth. However, in this exploratory study, the sample depth was relatively low, and care must be taken not to over interpret these preliminary results.

### Pointer year analysis

Most of the known historical drought years coincided with the negative pointer years in our four new *J. procera* chronologies (Fig. 8), confirming that pointer years can be used to independently verify the re-iterative cross-dating of *J. procera* samples. However, not all the narrow rings were drought induced (Eilmann et al. 2009) and not every pointer year could be directly associated with a drought (Fig. 8). This could reflect a lag in the negative effect of drought on tree growth which was supported by the relatively high autocorrelation in the site chronologies (Matisons et al. 2013). It has been demonstrated that *J. procera* is sensitive to climatic variability (Wils et al. 2010), and such trees show more pointer years than other tree species in the same area (Slimani et al. 2014).

## Conclusions

The sample preparation and cross-dating approaches employed in this study enabled the successful cross-dating of *Juniperus procera*. The detailed high-resolution observation of tree-ring anatomical features helped the identification of false rings, which aided cross-dating. Bomb-peak accelerator mass spectrometry radiocarbon dating was used to check the initial ring-width chronologies and revise after re-examining the ring widths. Buras (2017) inferred that low EPS is often observed in shrubs or anatomical chronologies; our study shows that in some cases to this list, chronologies from tropical trees should be included. Pointer years associated with historically recorded drought were identified and used to check the dating of the final chronology. This study confirms that *J. procera* growing in the Gonder region with unimodal precipitation has annual tree-rings and can be used to reconstruct historical variability in precipitation.

## Electronic supplementary material

ESM 1 (DOCX 18 kb)
