# Supplementary material for: Historical droughts recorded in extended Juniperus procera ring-width chronologies from the Ethiopian Highlands
Source: Int J Biometeorol. 2020 Feb 1;64(5):739–53. doi: 10.1007/s00484-020-01863-7 (PMC7220890; doi:10.1007/s00484-020-01863-7)
Supplement: Supplementary file 1 — (DOCX 18 kb) [file 484_2020_1863_MOESM1_ESM.docx]

**Supplementary table 1**

Presence and correspondence of historical drought years as negative pointer years in the four-site chronologies developed in this study. “*Neu”* – zero value of a pointer year statstics; “*Neg”* – negative pointer years “*Dry & Neg”* – negative pointer year corresponding drought year and “NA” - not available.

| Historical drought years | Gonder Qusquam church | Dabat Degua-Kidanemihret church | Woken Woybila Mariam church | Rise Adbarat Azezo Tekle haimanot church |
| --- | --- | --- | --- | --- |
| 1758 | Neg | NA | NA | NA |
| 1760 | Neg | NA | NA | NA |
| 1764 | Neg | NA | NA | NA |
| 1768 | Neg | NA | NA | NA |
| 1773 | Neg | NA | NA | NA |
| 1774 | Neg | NA | NA | NA |
| 1778 | Neg | NA | NA | NA |
| 1783 | *Dry & Neg* | NA | NA | NA |
| 1789 | Dry & Neg | Dry & Neg | NA | NA |
| 1794 | Neg | Neu | NA | NA |
| 1796 | Dry & Neg | Neu | NA | NA |
| 1797 | Dry & Neg | Neu | NA | NA |
| 1800 | Dry & Neg | Neg | NA | NA |
| 1808 | Neg | Neu | NA | NA |
| 1812 | Neg | Neg | NA | NA |
| 1819 | Neu | Neg | NA | NA |
| 1820 | Neg | Neu | NA | NA |
| 1822 | Neu | Neg | NA | NA |
| 1826 | Dry & Neg | Dry & Neg | NA | NA |
| 1829 | Dry & Neg | Dry & Neg | NA | NA |
| 1835 | Dry & Neg | Dry & Neg | NA | NA |
| 1837 | Dry & Neg | Dry & Neg | NA | NA |
| 1840 | Neg | Neu | NA | NA |
| 1846 | Neg | Neg | NA | NA |
| 1850 | Neg | Neu | NA | NA |
| 1853 | Neu | Neg | NA | NA |
| 1857 | Neu | Neg | NA | NA |
| 1860 | Neg | Neg | NA | NA |
| 1864 | Neu | Neu | Neu | Neg |
| 1867 | Neg | Neu | Neu | Neu |
| 1868 | Neu | Neu | Neu | Neg |
| 1870 | Neu | Neg | Neu | Neu |
| 1871 | Neg | Neu | Neu | Neu |
| 1872 | Neu | Neu | Neu | Neg |
| 1877 | Neu | Neg | Neu | Neu |
| 1878 | Neu | Neu | Neg | Neu |
| 1879 | Neu | Neu | Neg | Neg |
| 1880 | Dry & Neg | Dry & Neg | Dry & Neg | Dry & Neg |
| 1881 | Dry & Neg | Dry & Neg | Dry & Neg | Dry & Neg |
| 1885 | Neg | Neg | Neg | Neg |
| 1887 | Neg | Neg | Neg | Neg |
| 1889 | Dry & Neg | Dry & Neg | Dry & Neg | Dry & Neg |
| 1891 | Dry & Neg | Dry & Neg | Dry & Neg | Dry & Neg |
| 1892 | Dry & Neg | Dry & Neg | Dry & Neg | Dry & Neg |
| 1894 | Dry & Neg | Dry & Neg | Dry & Neg | Dry & Neg |
| 1899 | Neu | Neu | Dry & Neg | Dry & Neg |
| 1902 | Dry & Neg | Dry & Neg | Dry & Neg | Dry & Neg |
| 1910 | Neu | Neg | Neg | Neg |
| 1913 | Dry & Neg | Dry & Neg | Dry & Neg | Dry & Neg |
| 1914 | Dry & Neg | Dry & Neg | Dry & Neg | Dry & Neg |
| 1918 | Dry & Neg | Neu | Dry & Neg | Dry & Neg |
| 1922 | Dry & Neg | Dry & Neg | Dry & Neg | Dry & Neg |
| 1929 | Dry & Neg | Dry & Neg | Dry & Neg | Dry & Neg |
| 1931 | Dry & Neg | Dry & Neg | Neu | Dry & Neg |
| 1932 | Dry & Neg | Dry & Neg | Dry & Neg | Dry & Neg |
| 1933 | Dry & Neg | Dry & Neg | Dry & Neg | Dry & Neg |
| 1934 | Dry & Neg | Dry & Neg | Dry & Neg | Dry & Neg |
| 1935 | Dry & Neg | Dry & Neg | Dry & Neg | Dry & Neg |
| 1939 | Neg | Neg | Dry & Neg | Dry & Neg |
| 1940 | Dry & Neg | Dry & Neg | Neu | Neu |
| 1958 | Neu | Dry & Neg | Neu | Dry & Neg |
| 1965 | Dry & Neg | Neu | Dry & Neg | Dry & Neg |
| 1970 | Dry & Neg | Neu | Dry & Neg | Dry & Neg |
| 1971 | Dry & Neg | Dry & Neg | Dry & Neg | Neu |
| 1972 | Dry & Neg | Dry & Neg | Dry & Neg | Dry & Neg |
| 1973 | Dry & Neg | Dry & Neg | Dry & Neg | Dry & Neg |
| 1975 | Dry & Neg | Dry & Neg | Dry & Neg | Dry & Neg |
| 1976 | Dry & Neg | Dry & Neg | Dry & Neg | Dry & Neg |
| 1977 | Neu | Neu | Neu | Neu |
| 1978 | Dry & Neg | Dry & Neg | Dry & Neg | Dry & Neg |
| 1982 | Dry & Neg | Dry & Neg | Dry & Neg | Dry & Neg |
| 1984 | Dry & Neg | Dry & Neg | Dry & Neg | Dry & Neg |
| 1985 | Dry & Neg | Dry & Neg | Dry & Neg | Dry & Neg |
| 1990 | Dry & Neg | Dry & Neg | Dry & Neg | Dry & Neg |
| 1994 | Dry & Neg | Dry & Neg | Dry & Neg | Dry & Neg |
| 1995 | Neu | Neu | Dry & Neg | Dry & Neg |
| 2000 | Dry & Neg | Dry & Neg | Dry & Neg | Dry & Neg |
| 2005 | Dry & Neg | Dry & Neg | Dry & Neg | Dry & Neg |
| 2009 | Dry & Neg | Dry & Neg | Dry & Neg | Dry & Neg |
| 2012 | Dry & Neg | Neu | Neu | Neu |
